# CD133/Prominin-1-Mediated Autophagy and Glucose Uptake Beneficial for Hepatoma Cell Survival

**DOI:** 10.1371/journal.pone.0056878

**Published:** 2013-02-20

**Authors:** Haiyang Chen, Zaili Luo, Liwei Dong, Yexiong Tan, Jiamei Yang, Gensheng Feng, Mengchao Wu, Zhong Li, Hongyang Wang

**Affiliations:** 1 International Cooperation Laboratory on Signal Transduction, Eastern Hepatobiliary Surgery Hospital, The Second Military Medical University, Shanghai, China; 2 Department of Surgery, Eastern Hepatobiliary Surgery Hospital, The Second Military Medical University, Shanghai, China; 3 Department of Pathology, and Division of Biological Sciences, University of California San Diego, La Jolla, California, United States of America; 4 The 3^rd^ Affiliated Hospital and Medical College, Zhengzhou University, Zhengzhou, China; 5 State Key Laboratory of Oncogenes and related Genes, Shanghai Cancer Institute, Jiaotong University School of Medicine, Shanghai, China; The University of Hong Kong, Hong Kong

## Abstract

CD133/Prominin-1 is a pentaspan transmembrane protein that has been frequently used as a biomarker for cancer stem cells, although its biological function is unclear. The aim of our study was to explore the intrinsic functions of CD133 membrane protein in hepatoma cells during autophagy, apoptosis, tumorigenesis and cell survival through expression or downregulation of CD133. In this study, CD133 was found to be dynamically released from plasma membrane into cytoplasm in both of complete medium(CM) and low glucose medium (LGM), and LGM promoted this translocation. Expression of CD133 enhanced autophagic activity in LGM, while silencing CD133 attenuated this activity in HCC LM3 and Huh-7 cells, suggesting that CD133 is associated with autophagy. Immunofluorescence and time-lapsed confocal techniques confirmed that CD133 was associated with autophagy marker, microtubule-associated protein light chain3 (LC3) and lysosome marker during the glucose starvation. We further found that Huh-7 cells with stable expression of shCD133 (Huh-7sh133) impaired the ability of cell proliferation and formation of xenograft tumors in the NOD/SCID mice. Although loss of CD133 did not affect the rates of glucose uptake in Huh-7con and Huh-7sh133 cells under the CM, Huh-7sh133 cells obviously died fast than Huh-7con cells in the LGM and decreased the rate of glucose uptake and ATP production. Furthermore, targeting CD133 by CD133mAb resulted in cell death in HepG2 cells, especially in the LGM, via inhibition of autophagic activity and increase of apoptosis. The results demonstrated that CD133 is involved in cell survival through regulation of autophagy and glucose uptake, which may be necessary for cancer stem cells to survive in tumor microenvironment.

## Introduction

CD133, also called Prominin-1, has been used as a valuable marker for identification of normal stem cells, progenitor cells, and tumor initiating cells or cancer stem cells (CSC) [1]. Although CD133 expression has been detected in both differentiated and undifferentiated cells, CD133^+^ hepatocellular carcinoma cells exhibit stem-like properties in both *in vitro* and *in vivo* experiments, such as generating a xenograft that histologically resembles the parent tumor, the ability to self-renew, the capability to generate daughter cells that possess some proliferative capacity [2]–[6]. Ma et al. first identified the presence of 1.3% to 13.6% of CD133^+^ cells in 35 individual HCC specimens by flow cytometry that generated tumors in SCID/Beige mice in serial transplantations [7]. CD133-positive population is generally in a relative constant percentage in cell lines and tissues but increased in malignant transformation, which suggest that the transmembrane pentaspan protein may play an essential role in cell metabolism and survival [8]–[10]. Characterizing CD133 functions in tumor and incorporating these findings into cancer drug discovery might lead to better therapeutic approaches [11].

Accumulating evidence shows that the pentaspan CD133 protein is involved in a variety of cellular activities. CD133 is found to be selectively localized in microvilli and other plasma membrane protrusions irrespective of cell type [12]–[14]. Loss of CD133 causes disk dysmorphogenesis and photoreceptor degeneration [15]. CD133 specifically interacts with membrane cholesterol [12]. Hypoxic condition and mitochondrial dysfunction induces a reversible CD133 expression in human glioma, suggesting that CD133 mat be associated to bioenergetic stress [16]. Its expression is regulated by Wnt, Notch, TGFβ1, Line-1 and methylation [17]–[20]. BMP4 promotes CD133^+^ HCC CSC differentiation and inhibits their self-renew, chemotherapeutic resistance and tumorigenic capacity [21]. MiR-130b preferentially up-regulated in the CD133^+^ liver CSC cells via suppression of 53-inducible protein 1 [7], while miR-150 reduces CD133^+^ cells through downregulation of c-Myb proteins in HCC cells [22]. High expression of IL-8 in CD133^+^ liver tumor-initiating cells promotes angiogenesis, tumorigenesis, and self-renewal through neurotensin and MAPK signaling pathway [23]. Transcription factor AF4 was found to be a promoter of CD133 in multiple cancer cell lines [24]. In addition, CD133 has been found to be involved in endocytic-exocytic pathway [25] and transferrin uptake [8]. Targeting CD133 by its specific antibody leads to an inhibition of cell proliferation [26]–[28]. Treatment of CD133^+^ HCC cells with doxorubicin and fluorouracil significantly enriches the CD133^+^ subpopulation [29]. Gamma-irradiation of CD133+ glioma cells induced autophagy responsible for the resistance that can be inhibited by the autophagy inhibitor [30]. These results suggest that CD133-mediated regulation may be required for cell survival and stemness properties.

To determine the underlying mechanisms that CD133 is involved in maintenance and survival of hepatoma, in this study, we used several hepatoma cell lines to observe the roles of CD133 in membrane translocation, autophagy, proliferation, survival under the glucose starvation and xenograft tumor formation. CD133 was able to translocate from membrane to cytoplasm, promoted formation of autophagosomes under glucose starvation, and promoted glucose uptake and ATP synthesis, while knocking down CD133 reversed these activities and reduced xenograft tumor formation. Our data first demonstrates that CD133 is involved in autophagy which is beneficial for cell survival and tumor growth.

## Materials and Methods

### Cell Lines, Cell Culture, CD133 Expression and shRNA Plasmid and Lentivirus

LO2 cells (Chinese Academy of Science, Shanghai Cell Library, China) [31], HepG2 and Huh7 cell line (American Type Culture Collection), LM3 cells was a gift from Dr Lunxiu Qin(Zhongshan Hospital, Fudan University, Medical University) [32] were cultured in complete DMEM medium with high glucoses (4 g/L) or low glucose (1 g/L) (Invitrogen) containing 10% FBS in a humidified atmosphere with 5% CO2 and 95% air at 37°C. CD133 was amplified from cDNA of Huh-7 cells by RT-PCR and inserted into modified pCDH-CMV-Flag-GFP-puro (Bioscience, USA) by Nhe I/Hind III. CD133 shRNA were constructed into pSuper-EGFP vector according to previously described sequence [26]. Cherry- and GFP-CD133 were constructed in the backbone of pEGFP-N1. Lentivirus-tdTomato-shCD133 (5′- GCTCAGAACTTCATCACAAAC-3′) and control virus were prepared by 3DBiopharm, China.

### Transmission Electron Microscope

LM3 cells were plated in 10 cm dishes and transfected with p3xFlag-CD133 or empty vector, respectively. After 24 hours, the medium was changed with the LGM for 3 h. 1×10^7^ cells were harvested and fixed with 4% PFA for at least 6 hours and post-fixed in 0.5% osmium tetroxide for 30 minutes. The cells were then dehydrated in a graded ethanol series and mounted on a specimen stub. Sections were cut at a thickness of 80 nm and mounted on mesh copper grids and analyzed using Hitachi 7650 transmission electron microscope.

### Immunoblot Analysis

Cell lysates were separated in 7.5%, 10% or 15% SDS-PAGE gel. Proteins were transferred to the PVDF nitrocellular membranes and hybridized with primary antibodies for CD133/1(AC133, Miltenyi), LC3(#4108, CST), beclin-1(#3495, CST), β-actin (SC-1616, Santa Cruz), GADPH(#5174, CST), Atg5 (#M153-3, MBL), Atg9A(#NB110-56893, Novus Biologicals), AMPK(#2603, CST), GSK3β(#9315, CST), Glut1(#2944-1, Epitomics), phospho-AMPK(#2535, CST), phosphor-GSK3β(#9336, CST), respectively. The secondary anti-mouse or rabbit antibody was conjugated with IRDye 700 (Rockland Immunochemicals). Intensity of the fluorescence was scanned by the Odyssey system (Li-Cor, Lincoln, NE) and analyzed by Image 5.0 software.

### Apoptosis

Apoptosis was analyzed by two methods. One was using an apoptosis kit (APPLYGEN). At the end of tests, plates were washed twice with phosphate-buffered saline (PBS), and fixed in 4% paraformaldehyde (PFA) for 10 minutes. 0.25 ml of the apoptosis staining buffer was added to each well and kept for 10 minutes at room temperature. Images was taken under OLYMPUS IX70 system. Cells with condensate, dissolved or broken nuclei were counted as apoptotic cells. Flow cytometry approach was also used for apoptosis. Briefly, cells were trypsinized and collected at the end of tests. 1×10^5^ cells were suspended in 1% FBS/PBS, and 3 ml of ice-cold ethanol was added by droplet and kept at 4°C overnight. 1 ml of Propidium iodide (PI) solution (50 µg/ml PI and 3.8 mM sodium citrate) was added to cells after washing twice with PBS and stained for 3 h at 4°C. cells were analyzed with Moflo-XDP flow cytometer.

### Immunofluorescence Staining and Confocal Microscope

Immunofluorescence assay was performed as described previously [33]. LM3 or Huh-7 cells were seeded onto cover slips in 12-well plate and cultured in 10% FBS/DMEM at 37°C overnight. Cells either underwent transfection or directly were incubated in the LGM for autophagy analysis. Primary antibodies were used: anti-LC3 and anti-CD133 antibodies. The second antibodies used for the staining were: Alexa Fluor488 (goat anti-mouse or rabbit), Alexa Fluor555 (goast anti-mouse or rabbit) (Invitrogen). After fluorescent dye loading and washing, slides were mounted with antifade reagent (Molecular Probes). Results were observed and taken by Leica Confocal microscope or Olympus 1×71 inverted microscopes with a QImaging Retiga 4000R digital camera driven by QCapture Pro 5.1 image capture software.

### Assessment of Autophagy and Dynamics of CD133 Membrane Proteins

Cells were transfected with CD133-Cherry or CD133-GFP together with GFP-LC3. The cells were observed and recorded under fluorescence microscope (Olympus IX70, Leica Confocal TCS-SP5) at indicated times. Five images were taken in each well. Cells with more than six green fluorescence puncta were quantified as positive cells for autophagy. Total puncta were also counted in the same number of cells.

### Detection of Proliferation by Cell Counting Kit 8(CCK8)

Cell proliferation was detected with CCK8 (Dojindo) according to the instruction. Briefly, at the end of tests, a mixture of 10 µl of the reagent and 90 µl media was replaced in each well of 96-well plate, and the plate was incubated for 1 h and measured the absorbance at 450 nm by the BioTek Gen5 systerm (BioTek, US).

### Detection of ATP Product in Cells

At the end of culture, 96-well culture plate was equilibrated at room temperature for approximately 30 minutes before added 100 µl of CellTiter-Glo ® Reagent(Promega) for cell lysis. Plate was then rotated for 2 minutes to induce cell lysis and incubated at room temperature for 10 minutes to stabilize luminescence signal. Luminescence was read with BioTek Gen5 system.

### Spheroid Formation and Tumor Xenograft Models

Spheoid formation was carried out in 96-well ultra low attach culture plates with DMEM/F12 (invitrogen, #11320-033), B27 serum-free complement (Invitrogen, #17504-044), Insulin (Sigma, solution, bovine, #I0516), bFGF (Invitrogen, #13256-029), EGF (Sigma, #E4269). Spheroids were observed under microscope and quantified within two weeks. Media was replace every three days. Five-week-old male NOD/SCID mice were maintained and cared according to the university guidelines and animal protocols was approved by the Ethics Boards of the Eastern Hepatobiliary Surgery Hospital. 1×10^6^ Huh-7con or Huh-7sh133 cells were subcutaneously injected into the back side of each mouse. Animals were sacrificed when tumors were visible about 1 cm^2^.

### Glucose Uptake

Cells were seeded in 6-well plates with 10% FBS/DMEM overnight. To measure glucose uptake, cells were incubated with complete or low glucose media containing 100 µM 2-[N-(7-nitrobenz-2-oxa-1,3-diazol-4-yl) amino]-2-deoxy-_D_-glucose (2-NBDG) (Invitrogen) for the indicated time points up to 9 h, washed three times with PBS, detached by 0.25% trypsin/DMEM and detected with Moflo-XDP flow cytometer. Data were analyzed using software.

### Statistical Analysis

All data are expressed in means ± SD. Data sets were analyzed by software GraphPad Prism 5A. The Student’s t-test and the X2 test were applied to determine statistical significance. The value of p<0.05 was considered significant.

## Results

### CD133 is Associated with Activity of Autophagy

**Figure 1:**
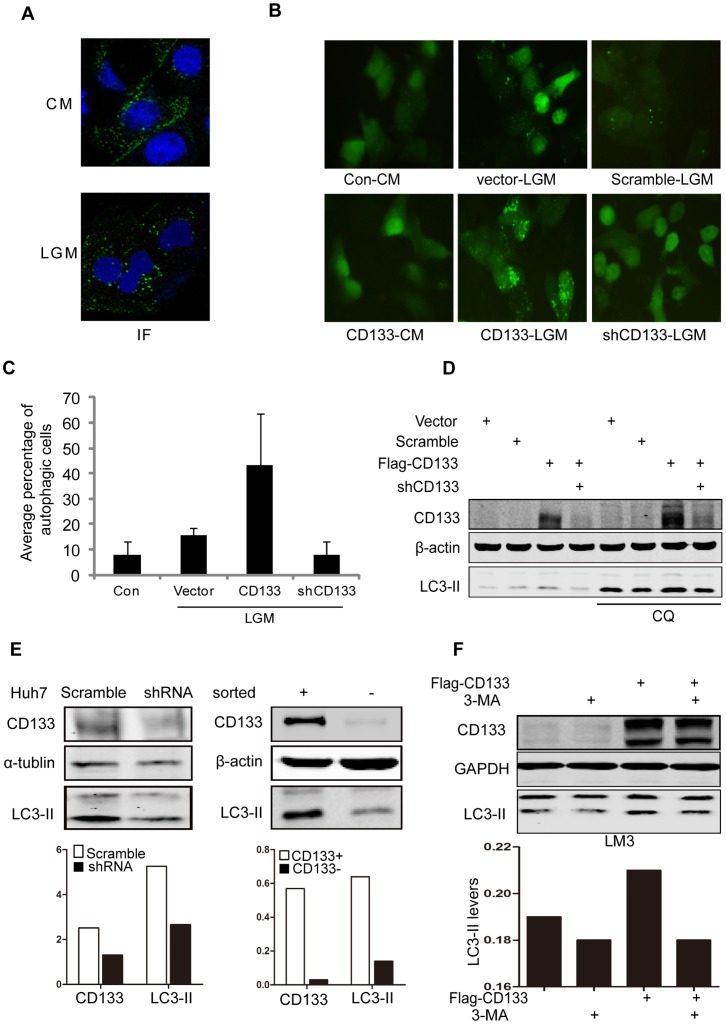
CD133 was released into cytoplasm and participated in autophagy. **A.** LGM condition promoted translocation of CD133 from membrane to cytoplasm. Huh-7 cells were stained by antibody for CD133 after culturing in CM and LGM for 3 h and counterstained by Hoechst. IF: Immunofluoresence. **B.** Autophagy in LM3 cells. Transient expression of plasmids as indicated together with GFP-LC3 in LM3 cells. Representative pictures were selected from observation at 1 h. **C.** Percentages of autophagic cells. Autohagic cells were measured by an individual cell with more than six puncta and expressed as mean±SD (n = 5). **D.** Levels of CD133 and LC3-II proteins in LM3 cells. p3xFlag-CD133 and pSuper-shCD133 were transfected into LM3 cells as indicated. Cells were harvested at 6 h after culturing in the LGM with or without 50 uM Chloroquine. **E.** CD133 levels were associated with LC3 levels. Under low glucose starvation for 3 h, silencing CD133 in Huh-7 cells reduced the level of LC3 (left). Isolated CD133^+^ huh-7 cells produced higher level of LC3-II than that in CD133^−^ Huh-7 cells (right). Densitometry analysis was done by normalization of CD133 and LC3-II levels with their own α-tubulin or β-actin and shown in graphs under immunoblots. **F.** Autophagy inhibitor abolished CD133-induced autophagy in LM3 cells. 3-MA (5 mM) inhibited CD133-induced increase of LC3-II in the LGM. Densitometry analysis was carried out by normalization of LC-II levels with GAPDH and shown under Western blotting panels.

CD133 has been found to be released from plasma membranes of hematopoietic stem and progenitor cells and multipotent mesenchymal stromal cells into media or/and form exsomes [25]. CD133 via clathrin and cholesterol-endocytosis inhibits tranferrin uptake [8]. These results indicate that CD133 may be dynamic along with changes of tumor microenvironment. First, we did immunofluorescence(ICC) for CD133 in Huh-7 cells. As shown in Fig. 1A, CD133 expressed on cell membrane in the CM condition, but there were tiny vesicles formed in cytoplasm close to membrane. However, upon starvation of cells in the LGM, the density of CD133 on membrane reduced dramatically and more fluorescence vesicles formed in cytoplasm (Fig. 1A), suggesting that CD133 may be released from membrane to cytoplasm.

Low glucose or nutritional deprivation triggers the autophagic activity. In process of autophagy, microtubule-associated protein light chain 3(LC3)conjugated with phosphatidylethanolamine becomes LC3-II association with both the outer and inner membranes of autophagosomes, which are visualized by fluorescence microscopy either as a diffuse cytoplasmic pool or as punctuate structures [34]. LM3 cells were transfected with p3xFlag-CD133 or pSE-shCD133 vector with GFP-LC3 in CD133^lo^ LM3 cells. In the CM, GFP-LC3 distributed evenly through cytoplasm and few pancta were formed in LM3 cells. In the LGM condition, more puncta formed in CD133-expressed cells than in vector-expressed control cells, while downregulation of endogenous CD133 expression by shRNA reduced the formation of autophagic puncta(Fig. 1B&C). Western blotting analysis showed that LC3-II level increased in forced expression of CD133 and reduced in silencing CD133. An inhibitor Chloroquince(CQ) for inhibition of lysosome-mediated degradation showed similar results (Fig. 1D), suggesting that CD133 is involved in endocytosis and autophagy. Because HCC Huh-7 cells have higher percentage of CD133 positive cells compared with other HCC cell lines, such as LM3 and HepG2 cells, we thus silenced CD133 in CD133^hi^ Huh-7 cells by shCD133 and meanwhile isolating CD133+ cells and CD133− cells from Huh-7 cells. Huh-7 cells with knocking down CD133 showed low activity of autophagy detected by the lower level of LC3-II compared to control Huh-7 cells with scramble vector (Fig. 1E left panel and below graph), and isolated CD133^+^ Huh-7 cells showed higher activity of autophagy than CD133^−^ Huh-7 cells in response to the low glucose stress at 3 h (Fig. 1E right panel and below graph). Treatment of LM3 cells with 3-methyladenine (3-MA), an autophagy inhibitor attenuated CD133-induced autophagy compared with control cells (Fig. 1F and Figure S1A). These results suggest that CD133 participates in autophagic activity in response to low glucose stress.

### CD133 Undergoes Autophagosomes and Lysosome Process

**Figure 2:**
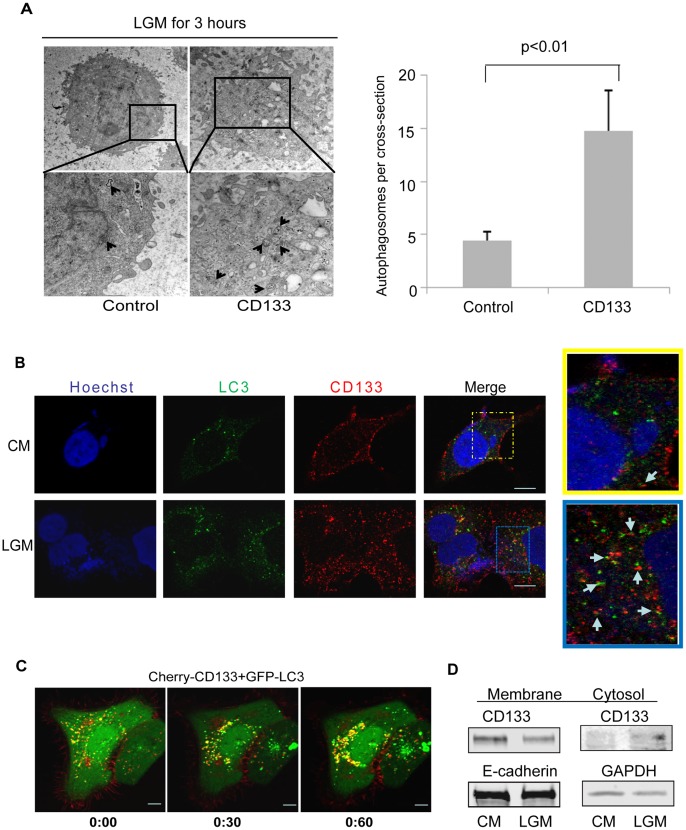
Expression of CD133 promoted autophagosome formation. **A.** LM3 cells were transfected with either empty vector or p3xFlag-CD133 and cultured in the LGM for 6 h and analyzed by transmission electron microscope. Arrows indicated autophagosomes. The number of autophagosomes per cell per cross-sectioned cells were counted (right graph) (mean±SD (n = 15). **B.** Huh-7 cells were placed on the coverslips overnight and cultured with fresh CM and LGM media for 3 h and subjected to confocal microscopic analysis. Magnified images (400X) are shown beside. Representative CD133-LC3 double-positive foci are indicated by arrows. CD133(red) and LC3(green). The scale bars represent 10 µm. **C.** CD133-Cherry and LC3-GFP were transfected into LM3 cells. Cells were observed with Leica confocal analytic system in EBSS. Images showed here were selected from a series of records (movie S1). The scale bar represents 10 µm. D. Western blotting showed CD133 levels in fragmentation of membrane or cytoplasm of cells after culturing in the LGM for 8 h. E-cadherin was as membrane control; GADPH as cytosol control.

To further determine the relationship between CD133 and autophagy, we detected autophagy in CD133-expressing LM3 cells and empty-vector control cells by transmission electron microscopy after low glucose stimulation for 3 h. Results showed that CD133-expressed cells exhibited more autophagosomes than control cells (Fig. 2A).

In order to observe the translocation of CD133, we stained CD133 and LC3 in Huh-7 cells in the CM and LGM condition, respectively. CD133 (red) was seen predominantly on cell membrane in the CM condition. There were some tiny vesicles in cytoplasm but CD133 was less co-localized with LC3(green) (Fig. 2B upper panels, and magnified images on upper right). However, in the LGM, intensity of CD133 on membrane reduced and there were more and bigger vesicles formed. CD133 staining associated with LC3 was clearly showed in a magnification (Fig. 2B lower panels and magnified image on lower right). Subsequently, the time-lapsed confocal fluorescence microscope recorded the movements of CD133 with LC3 in LM3 cells after transfection of CD133-Cherry and LC3-GFP. Fig. 2C were selected from the movies recorded within 1 h. The yellow-colored vesicles, referring as CD133-Cherry co-localized with LC3-GFP (Fig. 2C, see movie S1), increased in cytoplasm of LM3 cells. To determine the release of CD133 from membrane, we did that cytoplasm and membrane fragmentation of Huh-7 cells by ultracentrifugation after culturing in the CM and LGM for 6 h. Contents of CD133 reduced on membranes and increased in cytoplasm merely in the LGM (Fig. 2D). The results above confirmed that CD133 is involved in autophagy.

**Figure 3:**
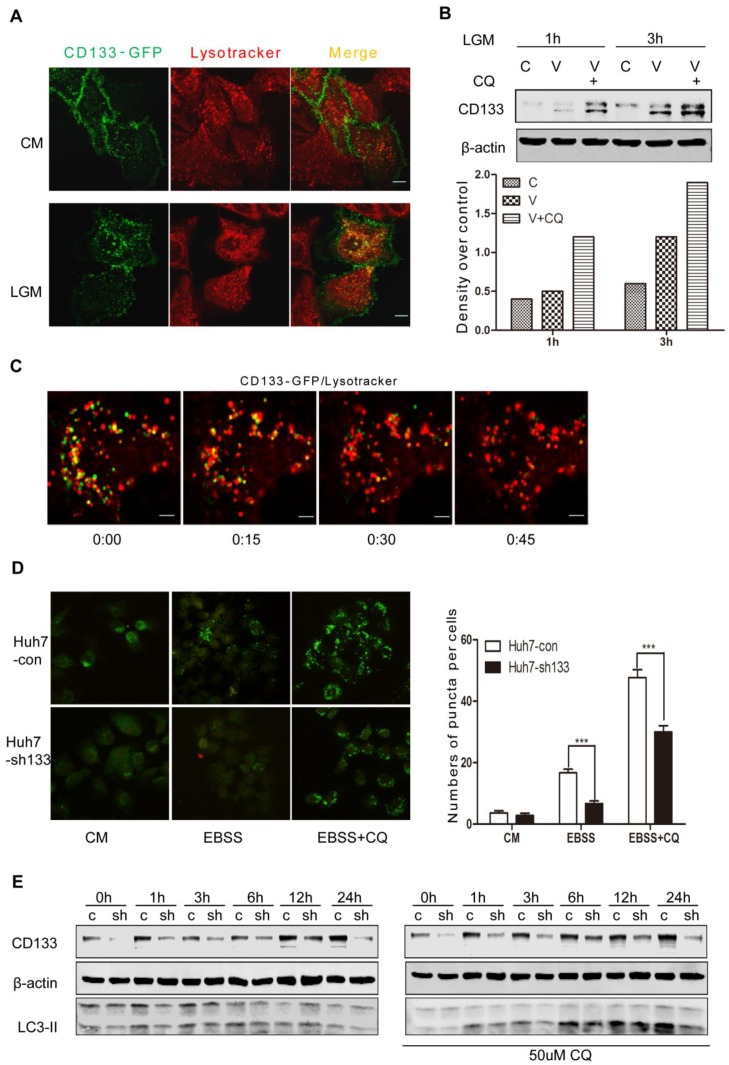
CD133 underwent lysosome degradation. **A.** LM3 cells on coverslips were transfected with CD133-GFP for 24 h and were labeled with Lysotracker for 1 h before observation. Localization of CD133 (green) and lysotracker (red) were observed under confocal microscope in the CM or LGM, respectively. The scale bars represent 10 µm. **B.** Inhibitory activity of lysosomes prevented degradation of CD133 in LGM. C: vector control; V: expression of CD133; CQ: Chloroquine. **C.** Movement of CD133 and lysotracker in LM3 cells. LM3 cells were transfected with CD133-GFP. Lysotracker(red) was added to cell medium for 1 h before observation with Leica confocal analytic system. Images showed here were selected from a series of records (movie S2). The scale bar represents 10 µm. **D.** The stable shCD133-expressing Huh-7 cell (Hun-7sh133) and con8trol cells(Huh-7con) were established by lentivirus infection and puromycin selection. Autophagy was observed after transfection of LC3-GFP into these cells. Images represented the situation of autophagy at 4 h in the CM, EBSS and EBSS+CQ conditions. Right graph showed number of puncta per cells. CQ: Chloroquine. **E.** Levels of LC3-II in these two cell lines at the time indicated after incubating in the LGM in presence or absence of chloroquine(CQ). c: Huh-7con cells; sh: Huh-7sh133 cells.

Formed autophagosomes eventually fuse with lysosomes for degradation of proteins, lipids and organelles. Thus, we traced CD133 and Lysotracker (Invitrogen) in LM3 cells by expression of CD133-GFP and then lysosome labeling by lysotracker. CD133-GFP expressed on cell membrane and lysotracker in cytoplasm under the CM condition. Noticeably, CD133 on membrane was reduced and colocolizations (orange) with lysotracker(red) were increased in cytoplasm upon glucose starvation (Fig. 3A), suggesting that CD133 proteins undergo degradation through lysosomes. This speculation was supported by an increase of CD133 level in presence of CQ (Fig. 3B). Meanwhile, process of CD133-GFP moving to lysotracker and disappearing in live cells under glucose starvation was observed and recorded under confocal microscope. CD133-GFP migrated and formed vesicles with red lysosomes labeled by lysotracker which became orange-colored vesicle and disappeared gradually at 45 min (Fig. 3C, see movie S2). Taken together, the results evidently demonstrated that membrane protein CD133 normally undergoes autophagy and lysosome degradation which were accelerated in response to the glucose starvation. Given that autophagy is a cellular protective response [35], we speculated that CD133 may be helpful for cell survival.

To better understand the role of CD133 in cell survival, we established cell lines with stable expression of CD133 RNAi (Huh-7sh133) or scramble RNAi (Huh-7con) using lentivirus carrying Tomato fluorescence and puromycin resistance. Consistently, GFP-LC3 distributed evenly in the cytoplasm of both Huh-7con and Huh-7sh133 cells in the CM, while LC3 positive puncta developed more in cytoplasm of Huh-7con cells compared to Huh-7sh133 cells, and Chloroquine inhibition of lysosomes stressed these effects (Fig. 3D left panels). Percentages of autophagic cells in Huh-7sh133 cells decreased significantly (Fig. 3D right graph). Parallel experiments without transfection of GFP-LC3 were used to detect the LC3-II levels in Western analysis. The results showed the similar results as observation under microscope (Fig. 3E).

### CD133-mediated Functions are Beneficial for Cell Survival

**Figure 4:**
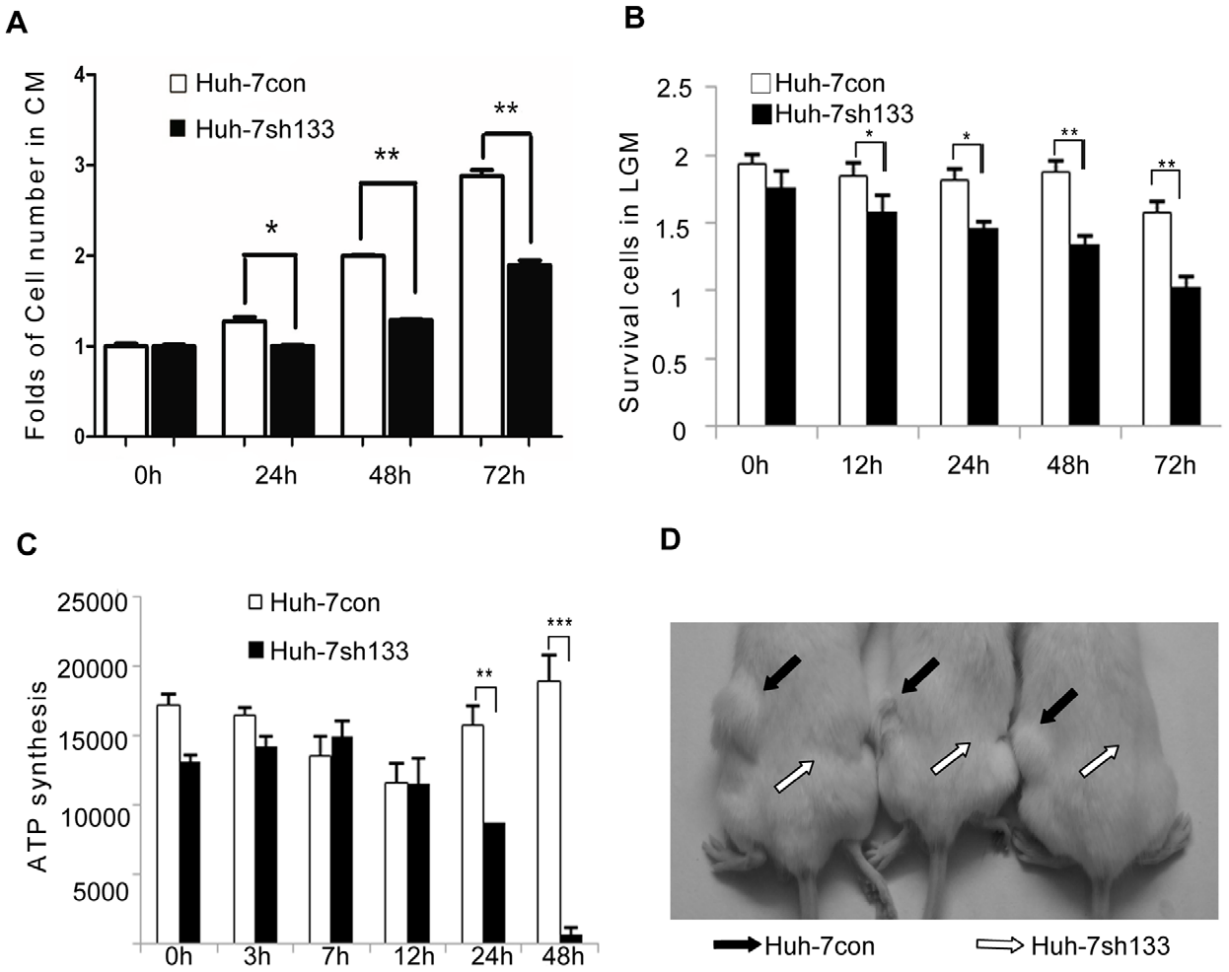
CD133-mediated autophagy was beneficial for cell survival. **A.** Silencing CD133 reduced proliferation of Huh-7 cells. Relative cell numbers were detected by CCK8 at the indicated time points. **B.** Huh-7con and Huh-7sh133 cells were cultured in the LGM for 3 days and cell numbers were detected by CCK8 at the time indicated (mean±SD, n = 3). **C.** ATP contents in Huh-7con and Huh-7sh133 cells in the LGM at during 2 days. **D.** 1×10^6^ cells from Huh-7con or Huh-7sh133 cells were subcutaneously injected into side back of NOD/SCID mice as indicated (3 mice). The significance of statistics is expressed as *p<0.05; **p<0.01; ***P<0.001.

To detect if CD133-mediated autophagy is involved in cell proliferation and survival, we analyzed the respective proliferation rates of Huh-7con and Huh-7sh133 cells in normal culture medium. Cell number was detected by CCK8 at 0, 24, 48 and 72 h and the folds of proliferation were obtained by the ratios of the value of each time over the one at 0 time respectively in each group. As shown in Fig. 4A, downregulation of CD133 expression significantly reduced the capacity of proliferation of Huh-7 cells. Because decrease of autophagic activity in glucose starvation, Huh-7sh133 cells were found dying faster than Huh-7control cells (Fig. 4B) in the LGM. Considering autophagy may be responsible for energy metabolism, we detected ATP synthesis by CellTiter-Glo ® Reagent(Promega). Silencing CD133 decreased ATP production compared to control cells (Fig. 4C). Meanwhile, Huh-7 cells with shCD133, CD133^−^ Huh-7 cells, and Huh-7sh133 stable cells failed to form spheroids in ultra low attach plates (Figure S1B) and xenograft tumors (0/3) in the immunodeficient NOD/SCID mice compared with control cells(3/3) (Fig. 4D).

### CD133 Promotes Glucose Uptake

**Figure 5:**
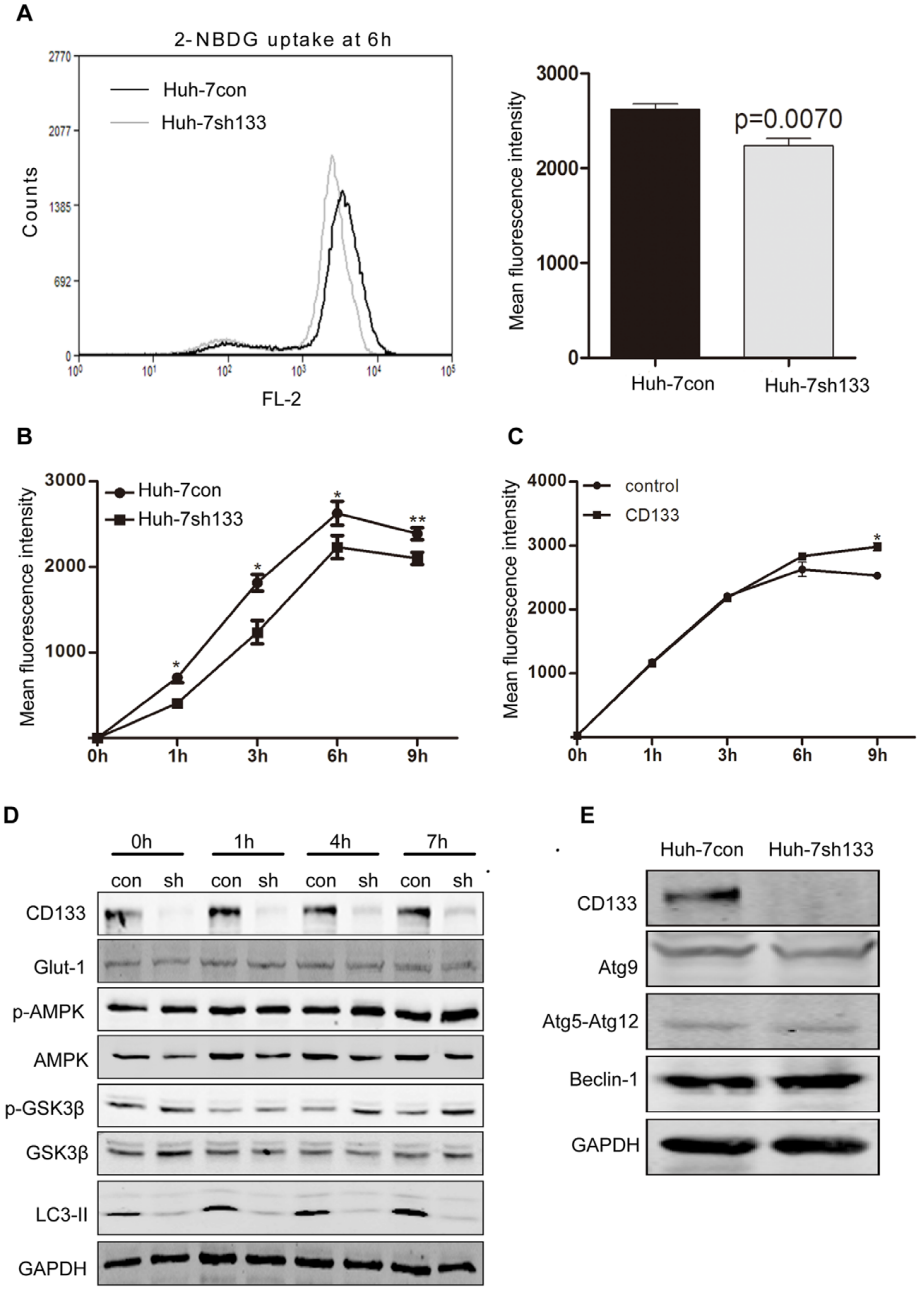
CD133 promoted glucose uptake. **A.** Levels of glucose uptake in Huh-7con and Huh-7sh133 cells. Mean fluorescence intensity of 2-NBDG uptake was measured by FASC analysis after incubating for 6 h. **B.** Time course of 2-NBDG uptake in these two cell lines. Statistical significance indicated as *p<0.05; **p<0.01. **C.** CD133 or empty vector was transfected respectively into LM3 cells. Glucose uptake was measured at the indicated times upon the medium added with 100 µM 2-NBDG. *p<0.05. **D.** Detection of relative activities of signaling pathways in Huh-7con and Huh-7sh133 cells by Western blotting. con: Huh-7con cells; sh: Huh-7sh133 cells. **E.** Detection of autophagic genes in Huh-7con and Huh-7sh133 cells by Western blotting.

To further determine the mechanism of CD133 in cell survival, in particular under nutritional starvation, we selectively detected the ability of glucose uptake in Huh-7con and Huh-7sh133 cells. These cells were respectively cultured in normal or low glucose medium containing 2-NBDG. Glucose uptake was detected by flow cytometry. Huh-7sh133 cells showed similar rates of glucose uptake as Huh-7con cells in the CM (data not shown), but Huh-7sh133 cells remarkably reduced glucose uptake in LGM (Fig. 5A&B). Overexpression of CD133 showed that LM3 cells increased glucose uptake constantly (Fig. 5C). These results suggest that CD133 is beneficial for cell survival possible through autophagy and glucose uptake.

Autophagy, a key homeostatic process of cytoplasmic degradation and recycling evolved to respond to stress conditions, is regulated by PI3K/TOR pathway and AMPK [36]. To determine if downregulation of CD133 would affect regulation of autophagy or autophagy-associated genes, we detected protein levels of these genes and their activities. The immunoblots showed that basal levels of AMPK and GSK3β appeared different in Huh-7con and Huh-7sh133 cells but became activated with similar tendency under the LGM condition. Although pAMPK and pGSK3β also increased in Huh-7sh133 cells, LC3-II levels were not elevated (Fig. 5D), suggesting that CD133 is important for autophagy. Furthermore, we confirmed that interference of CD133 did not affect expression of autophagy associated genes (Atg9A, Atg5 and Beclin-1) (Fig. 5E). These results indicate that CD133-mediated autophagy is not directly dependent on changes of expression of autophagy-associated proteins. Because of glucose transporter responsible for glucose uptake, we detected if silencing CD133 would affect the expression of glucose transporter type 1 (Glut1). The results showed that there were no differences in expression levels of Glut1 between these two kinds of cells (Fig. 5D). Therefore, our results first demonstrated that CD133 itself is beneficial for cell survival through promotion of autophagy and glucose uptake.

### CD133mAb-elicited Cytotoxicity through Inhibition of Autophagy and Increase of Apoptosis

**Figure 6:**
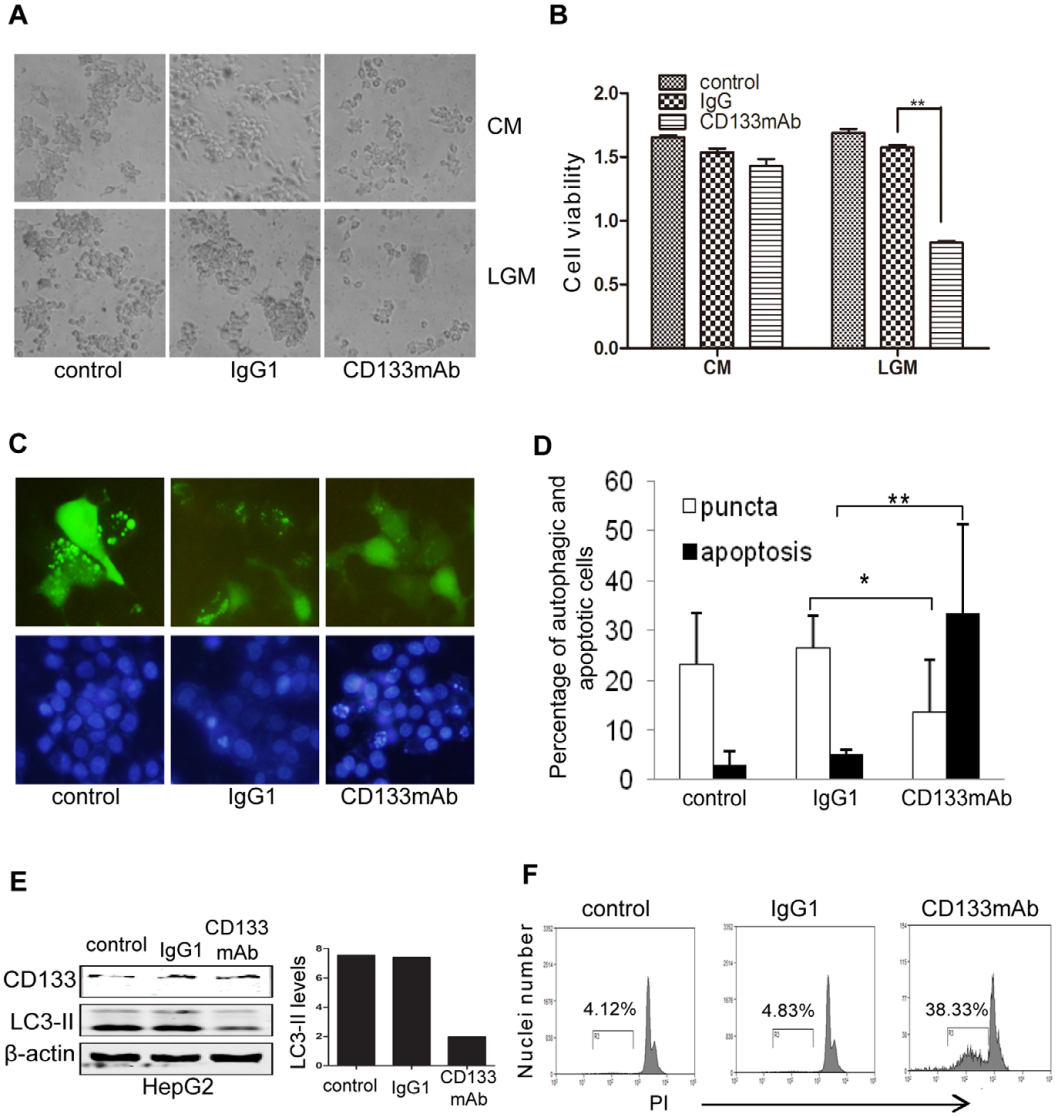
CD133mAb induced cell death through inhibition of autophagy and increase of apoptosis. A. HepG2 cells were treated with CD133mAb in the CM or LGM for three days. The images showed the situation at 48 h (magnification X200). Control: no treatment. IgG1∶1 µg/ml mouse IgG1; CD133mAb: 1 µg/ml AC133. **B.** Cell numbers were measured by CCK8 kit as described in Methods after treatment for 48 h(mean±SD, n = 3). **p<0.01. **C.** Effects of CD133mAb on autophagy and apoptosis of HepG2 cells. HepG2 cells were transfected with or without LC3-GFP for autophagy or apoptosis tests. The images (magnification X200) here showed the results of autophagy at 6 h after treatment (upper panels) and apoptosis (lower panels) at 48 h. **D.** Average percentages of autophagic or apoptotic cells were determined by five fields in each well and expressed as mean±SD. *p<0.05; **p<0.01. **E**. LC3-II levels in HepG2 cells after treatment for 48 h were analyzed by Western blotting. Normalization of LC3-II levels over β-actin was shown in right graph. The experiment was repeated at least three times. **F**. Apoptosis was detected in Huh-7 cells with flow cytometry. Histographs showed the distribution of DNA fragments in Huh-7 cells. Percentages were relatively number of cells with disrupted DNA.

It is known that CD133 antibody inhibits proliferation and colonization of cancer cells [26]. To understand the underlying mechanism, we examined effects of CD133 antibodies (AC133 and 293C2 Miltenyi, and #3663 Cell Signaling Technology) on HCC HepG2 cells. We found that antibody treatment did not show significant inhibition in CM (Fig. 6A upper panels) but strong inhibition occurred in LGM (Fig. 6A lower panels &B). These antibodies showed similar inhibitory effects (unpublished data), so we used AC133 referred as CD133mAb in this paper.

When treatment of HepG2 cells with control IgG1 and CD133mAb in LGM, we found that autophagic puncta were decreased in CD133mAb-treated HepG2 cells compared to untreated control and IgG1 treated cells (Fig. 6C, upper panels and Fig. 6D white bars). Western blotting showed a decrease of LC3-II in CD133mAb treated cells at 48h (Fig. 6E). These results suggest that antibody interacting with CD133 may attenuate CD133-mediated autophagy and elicit cell death. Apoptotic analysis showed an increase of condensed and broken nuclei in CD133mAb treated cells than those in IgG1 treated control (Fig. 6C lower panels & Fig. 6D black bars). Propidium iodide (PI) staining in HepG2 cells detected by flow cytometry further demonstrated that CD133mAb increased cell apoptosis up to 38% (Fig. 6F), while IgG1 showed little change the percentage of apoptosis cells compared with untreated cells at 24 h. The effects of CD133mAb on autophagy and apoptosis were also determined in Huh-7 cells and resulted in the similar results (unpublished). Taken together, these results indicate that CD133mAb-elicted cell death at least is through inhibition of autophagic activity and increase of apoptosis.

## Discussion

Since identification of CD133/prominin-1, a pentaspan membrane protein, there is a huge body of literatures addressing characterizations of CD133^+^ and CD133^−^ cells in normal and cancer cells [1]. However, biochemical mechanisms for membrane CD133 in cell regulation and protein-protein interaction remain to be elucidated. CD133 is selectively present in plasma membrane protrusions and apical microvilli irrespective of cell types [37], and enriched in the plasma membrane evaginations at the base of the outer segments of rod photoreceptors [12], [38]. Recently, CD133-containing membrane particles have been found to be released into various body fluids of adult humans, including saliva, seminal fluid and urine [25], [39]. These data suggest that CD133 trafficking in response to changes of cell microenvironment may be important for cell functions. In our study, we identified CD133 releasing from membrane into cytoplasm is involved in autophagy and promoted glucose uptake by which CD133 may function in cell survival. CD133 is reported to be associated with alteration of mitochondrial function in glioma cells [16] and cholesterol [40]. However, if CD133 also affects mitochondrial functions or cholesterol metabolism remains to be determined.

Autophagy is an evolutionarily conserved, intracellular self-protective mechanism for degradation of cytoplasmic material, damaged organelles and aggregate-prone proteins in lysosomes [41], [42]. It also plays a critical role in stem cell maintenance, self-renewal, and in a variety of cell differentiation processes [35], [43], [44]. Hypoxia/serum deprivation induces autophagy in mesenchymal stem cells [45]. Autophagy mediates survival of pancreatic tumor-initiating cells in a hypoxic microenvironment [46]. In agreement with the results that γ-irradiation induced a high level of autophagy in glioma CD133^+^ cells compared with CD133^−^ cells [30], we found that CD133 promotes autophagy which may be a possible mechanism underlying the resistance to irradiation and chemotherapy of CSCs [47]–[49]. Other data has shown that high expression of CD133 is associated with upregulation of ABCG5, or FLIP (caspase-8 inhibitor), and responsible for resistant to chemotherapy and apoptosis [50], [51]. Therefore, further study on mechanisms of CD133-mediated autophagy in chemoresistance or radiation would be necessary in HCC treatment.

Corbeil, Huttner and colleagues [25], [39], [52], [53] have revealed that CD133-containing membrane vesicles are released from neural progenitor cells or haematopoietic stem cells into the lumen of the neural tube or medium during differentiation, suggesting that these kind of membrane microdomains (lipid rafts) might host key determinants or players necessary to maintain stem cell properties [54]. In this paper, important finding is the identification of endocytic effect of CD133 in autophagosomes. The traffic of CD133 out or into cell membrane with lipids may be required for metabolism and signal transduction [55]. Although it is not yet known if CD133 is also associated with other functions, CD133 at least regulates autophagy and glucose uptake in response to low glucose stress for survival which may be one of reasons why CD133^+^ cells isolated from tumors have strong tumorigenicity [3], [56], and silencing CD133 abolished tumorigenecity of Huh-7 cells (Fig. 4D).

CD133 positive population can generate CD133 negative cells and CD133 negative cells may gradually generate CD133 positive cells in an in vitro culture system [57]. Dynamic change of CD133 membrane proteins are symbol for the alternation of tumor microenvironment such as the low glucose, hypoxia, and irradiation. Expression of CD133 may represent the need of self-renewal and undifferentiation [35]. Therefore, elucidation of CD133 bearing function is important for understanding stem/cancer stem cells. Targeting CD133 in HCC and other types of cancer may be better approach for elimination of CSCs through inhibition of CD133-linked signaling.

### Conclusions

In summary, CD133 is involved in autophagy and energy metabolism which are beneficial for the survival of cancer stem cells. Low glucose condition promotes CD133 antibody-induced cell death in HCC cells at least via blocking autophagy and increasing apoptosis. These results provide new evidence about the dynamics of CD133 membrane proteins necessary for energy process and cell survival in adaption to tumor microenvironment.

## Supporting Information

Figure S1 **A**. LM3 cells were transfected with p3XFlag-CD133 or empty vector together with LC3-GFP. After 24 h of expression, cells were incubated in the LGM in the presence or absence of 3-MA. Autophagy was then observed at 3, 6 and 12 h. The images were selected from the observation at 6 h. The puncta numbers were measured in five fields of each group and expressed as mean ±SD in right graph. **B**. Spheroid formation in CD133+ and CD133− hepatoma cells. Spheroid culture was applied to Huh-7 cells transfected with pSuper-GFP-shRNA-CD133 or pSuper-GFP-scramble vector(a), isolated CD133+ and CD133− Huh-7 cells(b), LM3 cells with expression of CD133 or vector(c), as well as Huh-7con and Huh-7sh133 cells(d). After 7–14 days, the spheroids in each group were showed in the left images and numbers of spheroids (over 20 cells) were measured in the right graph.
(TIF)

Movie S1 **CD133 was associated with LC3 under glucose starvation.** LM3 cells were seeded onto special culture chamber for microscope and transfected with CD133-Cherry (red) and LC3-GFP (green) vectors for 24 hours. Then cell medium was replaced with low glucose medium. Tracing and changes of two fluorescences were immediately recorded under Leica Confocal inverted microscope for 60 min. The picture was taken in every three minutes.
(WMV)

Movie S2 **CD133 was fused with lysosomes in the LGM.** LM3 cells were seeded onto special culture chamber for microscope and transfected with CD133-GFP (green) vector for 24 hours. Lysotracker (red) was added to the culture medium for 60 min. Then cell medium was replaced with low glucose medium. Tracing and changes of two fluorescences were immediately recorded under Leica Confocal inverted microscope for 45 min. The picture was taken in every three minutes.
(WMV)
