# Supplementary figures and images for: CD133/Prominin-1-Mediated Autophagy and Glucose Uptake Beneficial for Hepatoma Cell Survival
Source: PLoS One. 2013 Feb 20;8(2):e56878. doi: 10.1371/journal.pone.0056878 (PMC3577658; doi:10.1371/journal.pone.0056878)

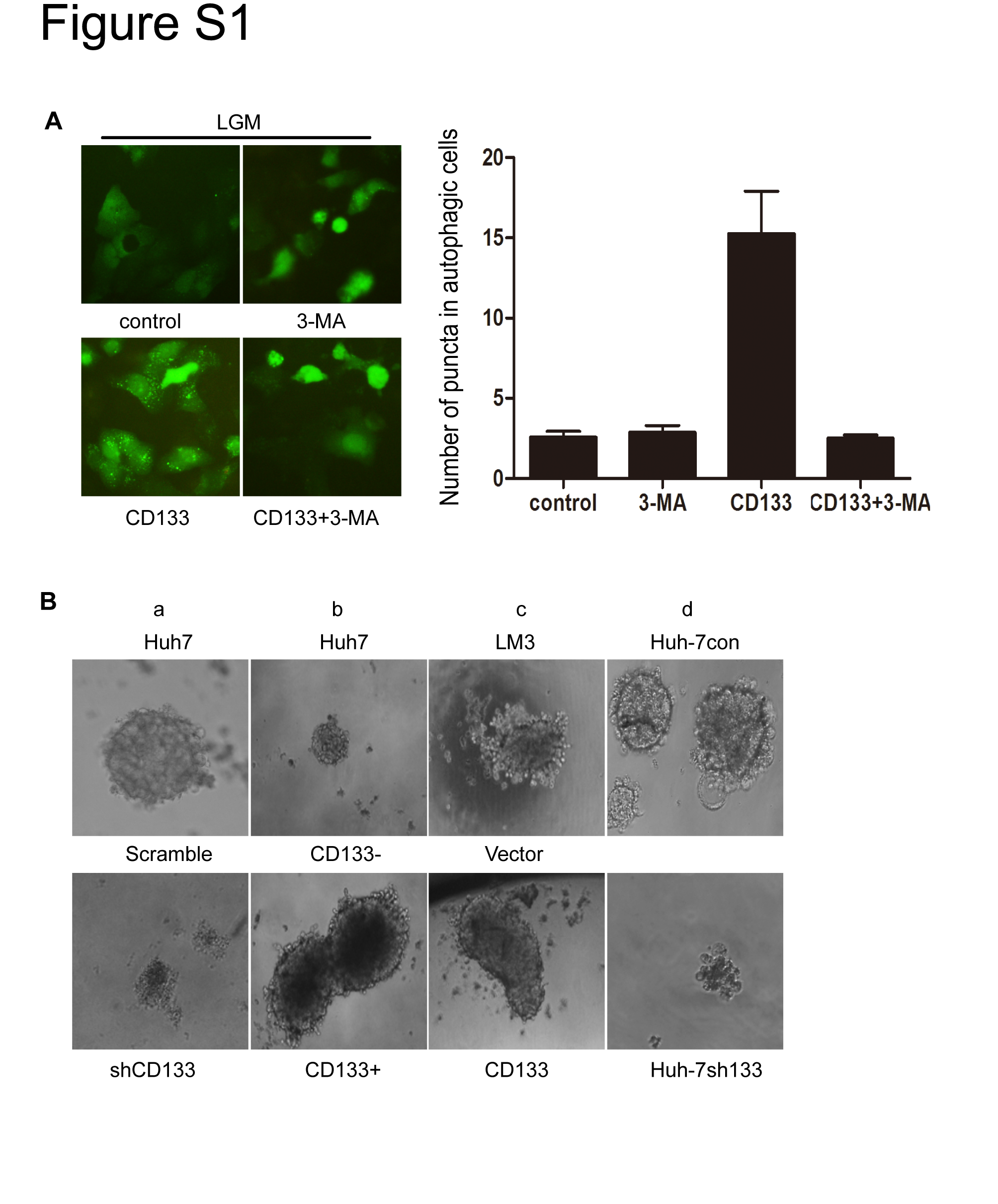

Supplement: Figure S1 — A. LM3 cells were transfected with p3XFlag-CD133 or empty vector together with LC3-GFP. After 24 h of expression, cells were incubated in the LGM in the presence or absence of 3-MA. Autophagy was then observed at 3, 6 and 12 h. The images were selected from the observation at 6 h. The puncta numbers were measured in five fields of each group and expressed as mean ±SD in right graph. B. Spheroid formation in CD133+ and CD133− hepatoma cells. Spheroid culture was applied to Huh-7 cells transfected with pSuper-GFP-shRNA-CD133 or pSuper-GFP-scramble vector(a), isolated CD133+ and CD133− Huh-7 cells(b), LM3 cells with expression of CD133 or vector(c), as well as Huh-7con and Huh-7sh133 cells(d). After 7–14 days, the spheroids in each group were showed in the left images and numbers of spheroids (over 20 cells) were measured in the right graph. (TIF) [file pone.0056878.s001.tif]
